# Supplementary material for: Reliability and Validity of the Single-Camera Markerless Motion Capture System for Measuring Shoulder Range of Motion in Healthy Individuals and Patients with Adhesive Capsulitis: A Single-Center Study
Source: Sensors (Basel). 2025 Mar 21;25(7):1960. doi: 10.3390/s25071960 (PMC11991502; doi:10.3390/s25071960)
Supplement: Supplementary file 1 [file sensors-25-01960-s001.zip › sensors-3515073-supplementary.pdf]

Supplementary Data

Measurement procedure

To measure the joint range of motion according to each shoulder joint movement, we will use the following instructions. Scapular rotation for each movement is permitted.

(A) Flexion: In an upright position, position your hands by your sides with your thumbs facing forward. Then, slowly raise your arms forward, keeping your elbows straight.

(B) Extension: In an upright position, position your hands by your sides with your thumbs facing forward. Then, slowly raise your arms backward, keeping your elbows straight.

(C) Abduction: Stand up straight and position your hands by your sides with your thumbs facing forward. Then, slowly raise your arms to the sides, keeping your elbows straight, with the backs of your hands pointing toward the ceiling.

(D) Adduction: Stand up straight and position your hands by your sides with your thumbs facing forward. Then, while keeping your elbows straight, slowly bring your arms inward.

(E) External rotation: Stand up straight and bend your elbows to 90°, with your thumbs facing forward. Place your upper arm next to your body, being careful not to move it, and move your lower arm outward with the back of your hand facing backward.

(F) Internal rotation: Stand up straight and bend your elbows at 90°, with your thumbs facing forward. Keep your upper arm next to your body, being careful not to move it, and move your lower arm inward.

(G) Passive abduction: In an upright position, the examiner holds the subject's shoulder joint with one hand and the subject's lower arm with the other hand, then slowly lifts the arm to the side, with the elbow extended.

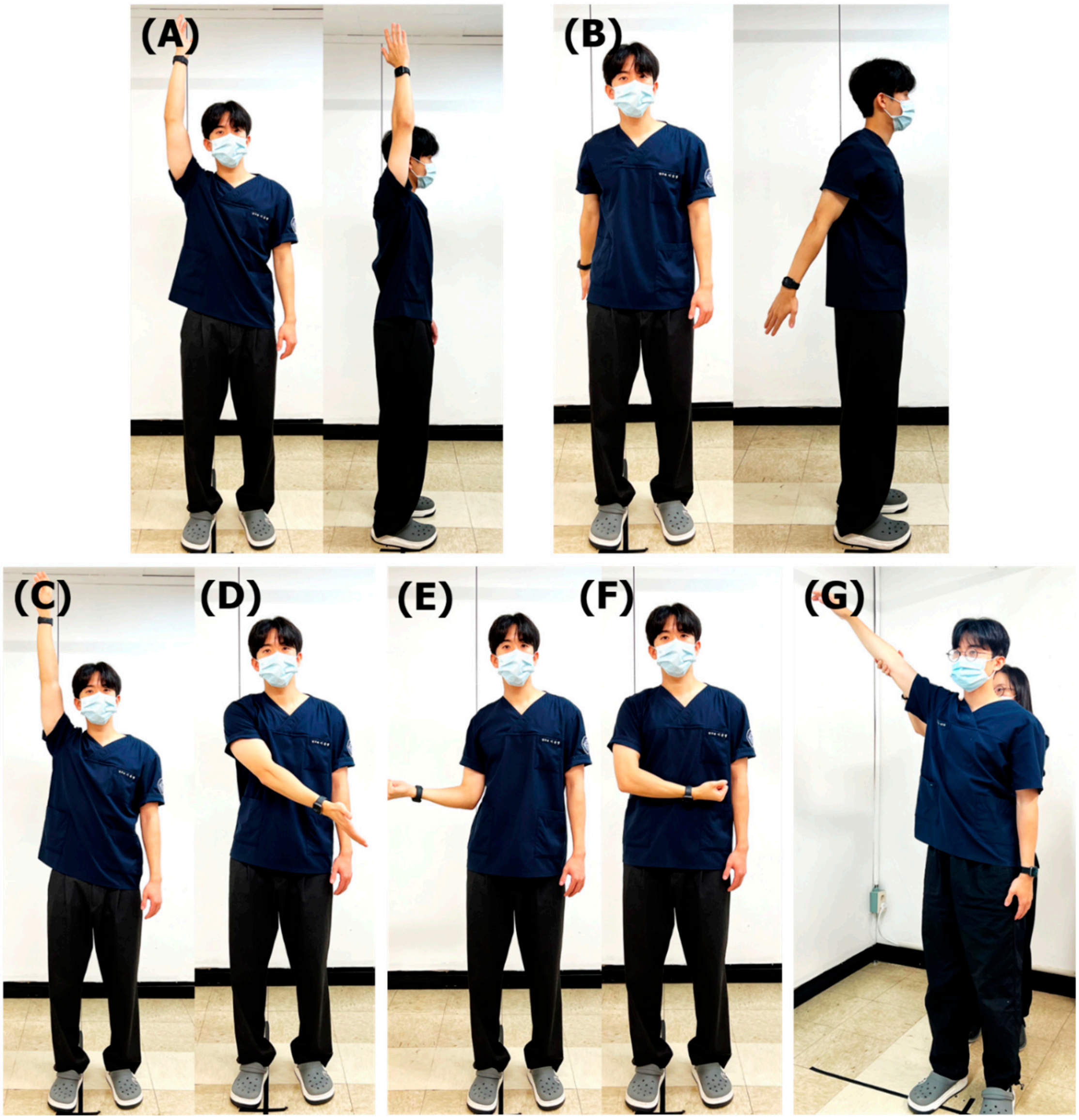

Figure S1. Supplementary Bland–Altman Plots for Additional Shoulder Movements: Extension, Adduction, Internal Rotation.

When performing measurements using a goniometer, the angle is measured in a two-dimensional plane. The details are defined as follows: flexion and extension angles are measured in the sagittal plane of the shoulder joint and are defined as the angles the arm makes in the coronal plane of the body. Abduction and adduction angles are measured in the coronal plane of the body and are defined as the angle between the arm and the sagittal plane of the shoulder joint. External and internal rotation angles are measured in an anatomical position with the elbow touching the torso and flexed to 90°. At this time, the external and internal rotation angles are defined as the angle between the sagittal plane of the shoulder joint and the arm, based on the transverse plane of the elbow. Passive abduction is measured by having the rater move the subject's arm to the point of mechanical limitation or pain.

Supplementary Tables

Table S1. Intraclass correlation coefficient (ICC) analysis for repeated measurements

Motion	ICC [2,1] (95% CI) - iBalance	ICC [2,1] (95% CI) - goniometer
Flexion	0.95 (0.94–0.96)	0.99 (0.99–1.00)
Extension	0.78 (0.74–0.82)	0.92 (0.90–0.94)
Abduction	0.99 (0.98–0.99)	0.99 (0.99–1.00)
Adduction	0.82 (0.78–0.85)	0.92 (0.88–0.94)
External rotation	0.81 (0.76–0.86)	0.97 (0.96–0.98)
Internal rotation	0.80 (0.76–0.83)	0.89 (0.87–0.92)
Passive abduction	0.98 (0.98–0.99)	1.00 (1.00–1.00)

Table S2. Reliability of goniometer for shoulder range of motion measurements

		ICC [2,1] (95% CI)	MAD	Margin 10%	p-value	SEM	MDC
Flexion	Inter-rater	0.95 (0.90–0.97)	4.05	16.55	<.0001*	3.75	10.40
	Intra-rater	0.95 (0.94–0.97)	3.74	16.58	<.0001*	3.63	10.05
Extension	Inter-rater	0.73 (0.62–0.80)	3.89	4.47	0.0109*	3.40	9.42
	Intra-rater	0.61 (0.49–0.71)	4.67	4.48	0.7382	4.07	11.29
Abduction	Inter-rater	0.97 (0.96–0.98)	3.11	16.69	<.0001*	4.02	11.13
	Intra-rater	0.98 (0.97–0.98)	3.16	16.74	<.0001*	3.68	10.19
Adduction	Inter-rater	0.77 (0.69–0.83)	4.00	3.71	<.0001*	3.60	9.99
	Intra-rater	0.64 (0.52–0.73)	5.28	3.72	0.0057*	4.34	12.02
External rotation	Inter-rater	0.88 (0.82–0.91)	4.30	7.68	<.0001*	4.05	11.22
	Intra-rater	0.85 (0.79–0.89)	4.90	7.69	<.0001*	4.42	12.26
Internal rotation	Inter-rater	0.71 (0.58–0.80)	3.56	8.13	<.0001*	3.28	9.10
	Intra-rater	0.60 (0.48–0.70)	4.13	8.14	<.0001*	3.82	10.58
Passive abduction	Inter-rater	0.99 (0.99–0.99)	1.50	16.88	<.0001*	2.22	6.16
	Intra-rater	0.99 (0.99–1.00)	1.70	16.93	<.0001*	2.05	5.70

p-values < 0.05 are marked with an asterisk (*). The ICC values were classified for reliability and validity based on the following criteria: excellent (0.75–1.00), good (0.60–0.75), fair (0.40–0.60), and poor (< 0.40). ICC, intraclass correlation coefficient; CI, confidence interval; MAD, mean absolute deviation; SEM, standard error of the mean; MDC, minimum detectable change.

Table S3. Reliability of iBalance for shoulder range of motion measurements based on hand dominance

			ICC [2,1] (95% CI)	MAD	Margin 10%	p-value	SEM	MDC
Flexion	Inter-rater	Dominant	0.95 (0.92–0.97)	3.39	16.72	<.0001*	3.61	10.01
		Right	0.94 (0.91–0.97)	3.39	16.75	<.0001*	3.61	10.02
		Left	0.91 (0.86–0.94)	3.42	17.24	<.0001*	3.72	10.32
	Intra-rater	Dominant	0.93 (0.89–0.95)	4.26	16.73	<.0001*	4.23	11.73
		Right	0.92 (0.88–0.95)	4.37	16.76	<.0001*	4.26	11.81
		Left	0.89 (0.83–0.93)	3.89	17.25	<.0001*	4.15	11.49
Extension	Inter-rater	Dominant	0.80 (0.69–0.87)	4.44	3.39	0.9799	4.27	11.83
		Right	0.79 (0.68–0.87)	4.60	3.37	0.9907	4.36	12.09
		Left	0.69 (0.53–0.80)	4.50	3.88	0.9019	4.16	11.52
	Intra-rater	Dominant	0.55 (0.36–0.70)	7.30	3.39	1.0000	6.36	17.62
		Right	0.55 (0.35–0.70)	7.48	3.36	1.0000	6.45	17.88
		Left	0.40 (0.17–0.59)	6.69	3.89	1.0000	5.86	16.26
Abduction	Inter-rater	Dominant	0.96 (0.93–0.97)	3.15	16.55	<.0001*	4.84	13.41
		Right	0.95 (0.92–0.97)	3.46	16.53	<.0001*	4.90	13.57
		Left	0.98 (0.96–0.99)	2.83	16.56	<.0001*	3.53	9.79
	Intra-rater	Dominant	0.91 (0.87–0.95)	5.48	16.50	<.0001*	6.76	18.74
		Right	0.91 (0.87–0.95)	5.41	16.53	<.0001*	6.73	18.66
		Left	0.96 (0.93–0.97)	3.79	16.68	<.0001*	4.53	12.56
Adduction	Inter-rater	Dominant	0.73 (0.59–0.83)	4.41	2.83	0.9995	4.02	11.13
		Right	0.73 (0.59–0.83)	4.55	2.83	0.9998	4.08	11.31
		Left	0.64 (0.44–0.77)	4.86	3.21	0.9990	4.40	12.19
	Intra-rater	Dominant	0.62 (0.44–0.75)	5.07	2.85	0.9997	4.84	13.42
		Right	0.69 (0.52–0.81)	4.77	2.85	0.9995	4.42	12.26
		Left	0.54 (0.34–0.70)	5.73	3.25	1.0000	4.99	13.84
External rotation	Inter-rater	Dominant	0.77 (0.52–0.90)	8.10	6.35	0.8418	7.96	22.05
		Right	0.77 (0.49–0.90)	8.70	6.32	0.8833	8.47	23.49
		Left	0.75 (0.57–0.87)	5.48	7.19	0.0248*	5.24	14.53
	Intra-rater	Dominant	0.39 (-0.10–0.73)	15.51	5.93	0.9996	12.61	34.96
		Right	0.40 (-0.12–0.76)	16.74	5.90	0.9997	13.35	37.01
		Left	0.63 (0.33–0.82)	7.06	7.12	0.4765	6.11	16.94
Internal rotation	Inter-rater	Dominant	0.71 (0.56–0.81)	4.36	7.41	<.0001*	4.19	11.61
		Right	0.68 (0.51–0.80)	4.41	7.35	<.0001*	4.23	11.73
		Left	0.77 (0.63–0.85)	4.32	7.19	<.0001*	3.98	11.02
	Intra-rater	Dominant	0.50 (0.28–0.67)	5.68	7.40	0.0066*	5.39	14.94
		Right	0.52 (0.30–0.69)	5.38	7.34	0.0014*	5.03	13.95
		Left	0.58 (0.40–0.72)	5.68	7.18	0.0069*	5.24	14.53
Passive abduction	Inter-rater	Dominant	0.96 (0.94–0.98)	4.23	16.51	<.0001*	4.89	13.55
		Right	0.96 (0.94–0.98)	4.13	16.61	<.0001*	4.86	13.48
		Left	0.98 (0.97–0.99)	2.88	16.76	<.0001*	3.02	8.36
	Intra-rater	Dominant	0.98 (0.96–0.98)	3.35	16.51	<.0001*	3.98	11.04
		Right	0.98 (0.96–0.98)	3.23	16.61	<.0001*	3.87	10.73
		Left	0.98 (0.97–0.99)	2.62	16.86	<.0001*	3.06	8.47

p-values < 0.05 are marked with an asterisk (*). The ICC values were classified for reliability and validity based on the following criteria: excellent (0.75–1.00), good (0.60–0.75), fair (0.40–0.60), and poor (< 0.40). ICC, intraclass correlation coefficient; CI, confidence interval; MAD, mean absolute deviation; SEM, standard error of the mean; MDC, minimum detectable change.

Table S4. Intraclass correlation coefficient (ICC) values for shoulder movements using iBalance in patients with adhesive capsulitis

	Inter-rater reliability	Intra-rater reliability	Validity
Flexion	0.81 (0.58–0.92)	0.77 (0.50–0.91)	0.50 (-0.10–0.80)
Extension	0.80 (0.56–0.92)	0.61 (0.23–0.83)	0.29 (-0.10–0.62)
Abduction	0.85 (0.66–0.94)	0.73 (0.43–0.89)	0.78 (0.59–0.88)
Adduction	0.66 (0.19–0.89)	0.48 (0.06–0.76)	0.31 (-0.08–0.61)
External rotation	0.82 (0.58–0.93)	0.27 (-0.18–0.65)	0.73 (0.52–0.85)
Internal rotation	0.69 (0.34–0.87)	0.06 (-0.42–0.67)	0.36 (-0.08–0.72)
Passive abduction	0.83 (0.62–0.93)	0.86 (0.68–0.95)	0.88 (0.71–0.94)

Table S5. Validity of iBalance for shoulder range of motion measurements compared to goniometer based on hand dominance

		ICC [2,1] (95% CI)	LOA	
			lower	upper
Flexion	Dominant	0.90 (0.83–0.93)	-5.74	17.22
	Right	0.90 (0.84–0.93)	-5.62	16.78
	Left	0.78 (0.29–0.91)	-4.52	19.03
Extension	Dominant	0.22 (-0.08–0.47)	-1.71	25.72
	Right	0.21 (-0.07–0.45)	-1.80	26.10
	Left	0.26 (-0.04–0.49)	-3.51	20.47
Abduction	Dominant	0.95 (0.93–0.96)	-6.16	17.14
	Right	0.95 (0.93–0.96)	-5.82	16.84
	Left	0.95 (0.93–0.97)	-5.80	15.14
Adduction	Dominant	0.39 (-0.06–0.66)	-2.78	19.25
	Right	0.39 (-0.04–0.65)	-3.07	19.32
	Left	0.39 (-0.07–0.67)	-1.68	19.12
External rotation	Dominant	0.82 (0.70–0.89)	-5.49	20.52
	Right	0.82 (0.70–0.90)	-5.84	21.32
	Left	0.58 (0.39–0.72)	-4.26	18.81
Internal rotation	Dominant	0.32 (-0.06–0.59)	-2.85	20.33
	Right	0.32 (-0.05–0.58)	-2.95	20.22
	Left	0.17 (-0.08–0.42)	-2.01	24.95
Passive abduction	Dominant	0.97 (0.95–0.98)	-4.93	14.11
	Right	0.97 (0.95–0.98)	-4.81	14.24
	Left	0.98 (0.96–0.99)	-3.46	10.21

p-values < 0.05 are marked with an asterisk (*). The ICC values were classified for reliability and validity based on the following criteria: excellent (0.75–1.00), good (0.60–0.75), fair (0.40–0.60), and poor (< 0.40). ICC, intraclass correlation coefficient; CI, confidence interval; LOA, limit of agreement; AC, adhesive capsulitis
